# COVID-19 Pandemic and Local Cultural Tourism in the Buzău Carpathians and Subcarpathians (Romania)

**DOI:** 10.3390/healthcare10122392

**Published:** 2022-11-29

**Authors:** Bianca Mitrică, Ines Grigorescu, Irena Mocanu, Paul-Răzvan Şerban, Nicoleta Damian, Monica Dumitraşcu, Cristina Dumitrică

**Affiliations:** 1 Human Geography and Regional Development Department, Institute of Geography, Romanian Academy, 023993 Bucharest, Romania; 2 GIS and Environmental Geography Department, Institute of Geography, Romanian Academy, 023993 Bucharest, Romania; 3 Physical Geography Department, Institute of Geography, Romanian Academy, 023993 Bucharest, Romania

**Keywords:** cultural tourism, COVID-19, tourists, businesses, residents, Buzău Carpathians, Subcarpathians, H2020 SPOT Project

## Abstract

The COVID-19 pandemic was and continues to be a major challenge for tourist activities worldwide. Cultural tourism was mostly hit because of the social distancing measures, travel restrictions and ban on people gatherings for cultural events. The current study is trying to provide an overall image of the challenges local cultural tourism has faced during the COVID-19 pandemic in a predominantly rural area of Romania—the Buzău Carpathians and Subcarpathians. The area has a high tourist potential stemming from the complexity and diversity of the natural landscapes and the local historical setting. The survey relies on two field campaigns carried out in 2020 when 161 questionnaires were applied to tourists, businesses and residents. The results highlight the significant impact of the COVID-19 pandemic on small businesses during and after the travel restrictions (e.g., revenue losses, temporary business shut-downs and layoffs/technical unemployment), but also on tourists’ travelling preferences and sentiment and on residents’ attitudes and behaviour.

## 1. Introduction

Tourism is one of the world’s major economic sectors, making up the third-largest export category (after fuels and chemicals) which accounted for 7% of global trade in 2019. Before the outbreak of the pandemic, the tourism sector accounted for 10% of the global GDP and employed approximately 320 million persons around the world [1]. In some countries, tourism can rise up to over 20% of the GDP, supporting 1 in 10 jobs and providing livelihoods for many millions more in both developing and developed economies [2]. It plays an enormously important role in the EU economy and society, generating foreign exchange, maintaining jobs and businesses, and driving local development and cultural exchanges [3]. Tourism remains a branch of consequence, whose development will be constantly in close correlation with the levels and rhythms of development of the other branches of the national economy [4]. The tourism industry is linked with every sector of the economy, but its development as a domain may, in some cases, be independent to other economic sectors [5]. Tourism and economic growth are also well connected, but there are so many factors which affect tourism positively and negatively [6]. Among these factors most cited are: accessibility and infrastructure [7,8,9], cost of staying, education [10,11], travel facilitation including easing of visa regulations [10], security [9,10,12], culture and language, natural and atrophic landscape [9,11], health pandemics and global financial crisis [12,13], income level [14], policy formulation and implementation [7,15], and attitudes of host communities [12]. Sustainable tourism development requires the informed participation of all relevant stakeholders, as well as strong political leadership to ensure wide participation and consensus building. Achieving sustainable tourism is a continuous process and it requires constant monitoring of impacts, introducing the necessary preventive and/or corrective measures whenever necessary [16].

The COVID-19 pandemic posed an immediate threat to the tourism industry and instantly influenced travel-related decision making.

Most of the world’s markets were faced with pandemic-related hardships, the scale of this crisis and its devastating effects on operations, employees and customers being unmatched by previous crises [17,18]. After the pandemic spread across the globe, its intensification and the related consequences have become dependent on a wider variety of factors, generating a system of complex interrelationships [19]. Thus, the COVID-19 pandemic had a much more significant impact on the global travel industry than the previous health crises because of a much greater geographical expansion, leading to the widespread implementation of travel bans and lockdowns affecting domestic travel [13,20], in general, and international travel, in particular. The pandemic has been acknowledged as an extreme outlier in terms of its implications for tourism [21], as it was and continues to be a challenge for the overall worldwide tourist activity, an unprecedented crisis for the tourism economy as a result of the immediate and immense shock it triggered [22,23,24,25], generating devastating effects for the tourism sector [26]. By the same token, the COVID-19 outbreak altered the tourism model worldwide [27] and has given rise to a new model of tourism governance [28], the shock being felt differently by entrepreneurs/businesses, tourists and residents.

Like the case of previous crises (e.g., SARS, Ebola, the global economic downturn) it is almost certain that recovery will follow. There are two questions—how long will the effects last, and when will the eventual recovery begin? These questions rise the need for research on the effects of such events, and the attitudes of those effected [29], namely entrepreneurs/business, tourists and residents. The impact of the perceived risks on travel behaviour and tourism decision making during and after the COVID-19 pandemic has already been identified in different studies by Fusté-Forné et al. (2021) [23], Gaffar et al. (2022) [25] and Matiza, 2020 [30].

Given that the current paper intends to approach cultural tourism in relation to the COVID-19 pandemic through the businesses’, tourists’, and residents’ behaviour, we highlight that businesses were among the most impacted components of the tourist activity within the larger context of the disruption of value chains and the decrease in international trade [31,32,33]. The economic collapse caused by the COVID-19 pandemic has been dramatic [34], the United Nations World Tourism Organization (UNWTO) estimating that 100–120 million direct tourism jobs were at stake, which included both low-skilled and high-skilled workers. Compared to the previously observed crises, the economic collapse caused by the COVID-19 pandemic has been more dramatic [34]. The results of the World Youth Student and Educational Travel Confederation survey on businesses revealed a downturn in demand in almost all travel sectors and world regions [29].

Strategies to decrease the COVID-19 trend, such as community lockdowns, social distancing, stay-at-home orders, travel and mobility restrictions, have led to the temporary shut-down of many hospitality businesses and to a significantly reduced business demand [35]. Taking the impact on closely linked sectors into account, the drop in international arrivals has caused an estimated loss of about 2.4 USD trillion in GDP in 2020 [36]. The 2020 declining trend in demand of almost all of the companies in the tourism industry [29] was followed by the same trend in 2021, which contributed to a deterioration of their business prospects [37]. Sectoral job losses have been prevalent, particularly in the most vulnerable host communities with their highly dependent tourism workers [38], the negative impacts felt being related to economic activity, unemployment, revenue loss and business shutdown [39]. Measures taken to combat the COVID-19 pandemic have harmed economic growth and have significantly restricted population movement, both inside and outside the country [40].

Pandemics, in general, have led to a significant decline in tourist arrivals. During the COVID-19 pandemic, in particular, supply, demand, spending and consumer confidence have all disintegrated [41]. Internationally, the number of overnight stays decreased over the 2019–2020 period by 74%, from 1.5 billion to 381 million, 2020 being the worst year on record for tourism [42]. There is a significant association between the past records in international tourism and the cumulated numbers of confirmed cases and deaths caused by COVID-19 [43]. In 2021, tourism experienced a 4% increase (i.e.,15 million more international tourist arrivals compared to 2020), but the international arrivals were still 72% below the pre-pandemic level of 2019 [42]. The health risk has become an important factor impacting tourists’ behaviour during the COVID-19 pandemic [17,44,45]. COVID-19 can be regarded as a health hazard for tourism, and the consumer’s perception of risk would be higher during a pandemic [46], having the strongest effect on a person’s intention to travel [47]. Risk perception influenced the perception of recreation site quality, the pandemic having a negative impact on visits [48]. Modern tendencies prove that there is a higher demand for the subsequent experiences: safe tourist destinations, in the most general sense of the concept of safety, including psychological, physical, food safety, and certifications deemed “clean and safe”; nature destinations; privacy, associated with less massified destinations; comfortable accommodation; less fractional and more extended vacations; and proximity [27].

Within the destination community, residents have a variety of reactions to the COVID-19 pandemic as it relates to tourism. It is difficult to predict resident attitudes toward tourism because of the complexity of community systems and their “disparate variables” [49]. There are multiple factors which influence a host community, such as the level of concern displayed by the community, its involvement in tourism, the community’s level of attachment, the development level of the local economy, the economic and social benefits, and the socio-cultural costs [50]. The perception of positive or negative impacts within the resident communities depends on the demographic factors (e.g., age, gender, income, education, location) [17]. Regardless of the economic benefits that tourists may bring, residents have expressed their concerns about the health risks that would accompany the arrival of tourists [51].

Cultural tourism, in particular, was mostly hit because of the social distancing measures, travel restrictions and the ban on people gatherings for cultural events. Heritage tourism, together with the cultural and social fabric of communities were the most affected by COVID-19 in terms of the postponement or closure of many intangible cultural heritage practices, i.e., traditional festivals, markets for handicrafts, products and other goods. World Heritage Sites were closed in 90% of countries having significant socio-economic consequences for tourism-reliant communities. Moreover, 90% of the museums were closed, 13% of which may never reopen [42]. As a consequence, the livelihoods of millions of cultural professionals have been seriously jeopardized [52]. According to Barchielli et al. (2022), young adults had higher scores in preoccupation, change of habits, and willingness to change habits, this group also reporting a fear of disease, its consequences, and the likelihood of isolation linked to COVID-19. Adults are the least worried, expressing less fear, and seem to be less willing to change their habits but they showed a fear of possible vaccine consequences, while elders have least changed their current habits being the most vulnerable to the disease, also being the most successful in limiting social contact [53]. In Romania, the situation was not very different. Despite the fact that the Romanian territory has a rich and valuable heritage potential with tourist attractions included on the map of European cultural routes [54], the country offers a great variety of cultural heritage to travellers in a short time, such as Byzantine churches, roman basilicas, gothic cathedrals, Turkish mosques, Greek fortresses, cubist or 1900’s style buildings [55,56], the effects of the COVID-19 pandemic were significant. In Romania, the patient zero infected with the SARS-CoV-2 virus was found on 26 February 2020 and one year later, on 24 February 2021, there were 788,048 cases of people infected with COVID-19 declared (the real numbers likely far surpass this figure due to insufficient testing [3,17]), 731,049 patients had been cured, and 20,287 were deceased. For the tourism industry, the year 2020, the most atypical year in the history of Romanian tourism, was full of trials and challenges [57]. According to the National Institute of Statistics, the number of overnight stays in Romania decreased by 51.5% over the 2020–2021 period, from 30.0 million to 14.5 million. This reflects the impact that the pandemic has had on Romanian tourism, in general, as well as on cultural tourism, in particular.

Concerning their effects on tourism, as well as on several adjacent economic activities, at least three major stages can be distinguished in Romania:

(1) Between 15 March and 15 May 2020, a general lockdown was put into place throughout the country. The imposed measures were very strict, and they mainly included the mobility of citizens, the transportation of goods, the limitation of public transport, sanitary measures, economic measures [58]. All these restrictions affected the national tourism industry, as well as the local tourism within the study area.
(2) November 2020, the Romanian Government took the measure of closing down all Romanian markets. The closing of fairs had important negative effects for tourist activities within the Buzău Carpathians and Subcarpathians, where, in 2019, 22 markets and fairs associated with cultural and/or religious events were organised [59]. They were all shut down in 2020.
(3) Between March and April 2021, when several sets of measures entered into force, such as the quarantine for different settlements (e.g., those surrounding the capital-city and other big cities, some large urban centres or small isolated localities) and periods of time (e.g., two weeks and/or during the weekends). The imposed quarantine measures restricted the free movement of people, thus making the tourists’ access to the tourist attractions impossible.

This research takes into account a series of investigations undertaken within the Horizon 2020 SPOT project (Work package 1: Data and documentation on cultural tourism, http://www.spotprojecth2020.eu/reportsandoutcomes, accessed on November 18 2022), where the perception of three main components of the tourism system in the Buzău Carpathians and Subcarpathians was analysed differently and comparatively: tourism businesses, tourists and residents. The aim of this research was to find out more about the similarities and differences that exist in entrepreneurs’, tourists’ and residents’ behaviour and the impacts of the COVID-19 pandemic on cultural tourism in Romania, and specifically in the Buzău Carpathians and Subcarpathians. Moreover, we were interested in analysing tourists’ experiences, as well as the residents’ and tourism entrepreneurs’ views during the pandemic and what the influence of COVID-19 had been on cultural tourism (e.g., experienced changes, discovered opportunities) in a rural area with a potential for cultural tourism nevertheless underdeveloped from this perspective. In this study, the authors are focused on filling this knowledge gap by performing a perception analysis focused on the businesses’, tourists’ and residents’ perspective on cultural tourism, relying on collecting data from the key informants in the case study area.

The research questions addressed are grouped in order to respond to the three categories of the parties interviewed: 

RQ1 Business: How the business has been affected, the measures taken to offset the negative impact, in general, and the relationship with the employees, in particular, the assistance received from the government and in order to sustain the business during the COVID-19 crisis:

RQ2 Tourists: How the COVID-19 crisis changed the way of travelling and how satisfied tourists were during visit(s) prior to and after the COVID-19 crisis.

RQ3 Residents: The relation between residents and cultural tourism by underlining the participation in cultural activities in their own surroundings prior to and during the COVID-19 crisis.

A Hypothesized Model aimed at better addressing the Research Questions was proposed. Studies have determined that the COVID-19 pandemic influenced tourists travel propensity (especially cultural tourism), local business and resident behaviour.

**Hypothesis 1 (H1).**  *The COVID-19 pandemic positively and significantly affects travel propensity. We have examined the tourist arrivals before and during the COVID-19 pandemic period. Travel restrictions, accommodation and limited restaurants capacity impacted travel propensity. Compared to 2019, tourists arrivals decreased by 60%*.

**Hypothesis 2 (H2).**  *The COVID-19 pandemic negatively and significantly affects local business. The lower number of tourist arrivals reduced the activity of tourism entrepreneurs.*

**Hypothesis 3 (H3).**  *The COVID-19 pandemic altered residents’ behaviour related to cultural activities participation. Within the COVID-19 pandemic context, residents have chosen to visit cultural sites and participate in cultural activities in their own surroundings less than before, as reflected by the field enquiry.*

## 2. Study Area

**Figure 1:**
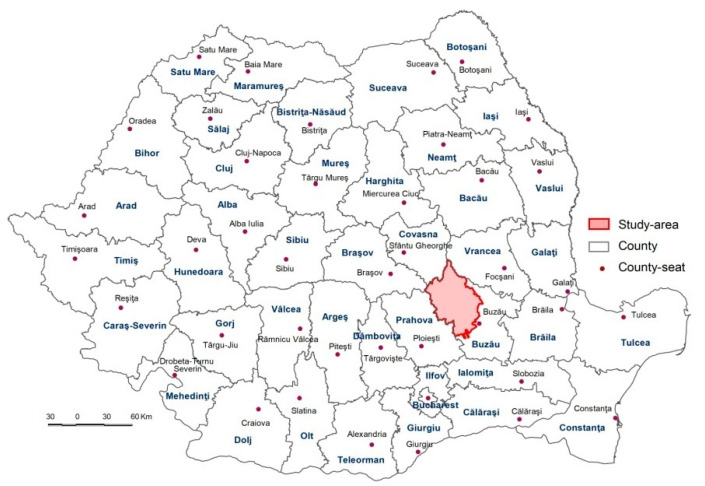
The location of the study area in Romania.

The Buzău Carpathians and Subcarpathians are located in Buzău County, within the South-East Development Region (Figure 1). The area consists of 34 Local Administrative Units (LAUs), two of which fall under the urban category (towns) and 32 under the rural one (communes). The study area accounts for around 128,000 inhabitants (2018), 86.1% of which are rural, and 13.9% are urban, and has a density of about 47.6 inh./km^2^.

**Figure 2:**
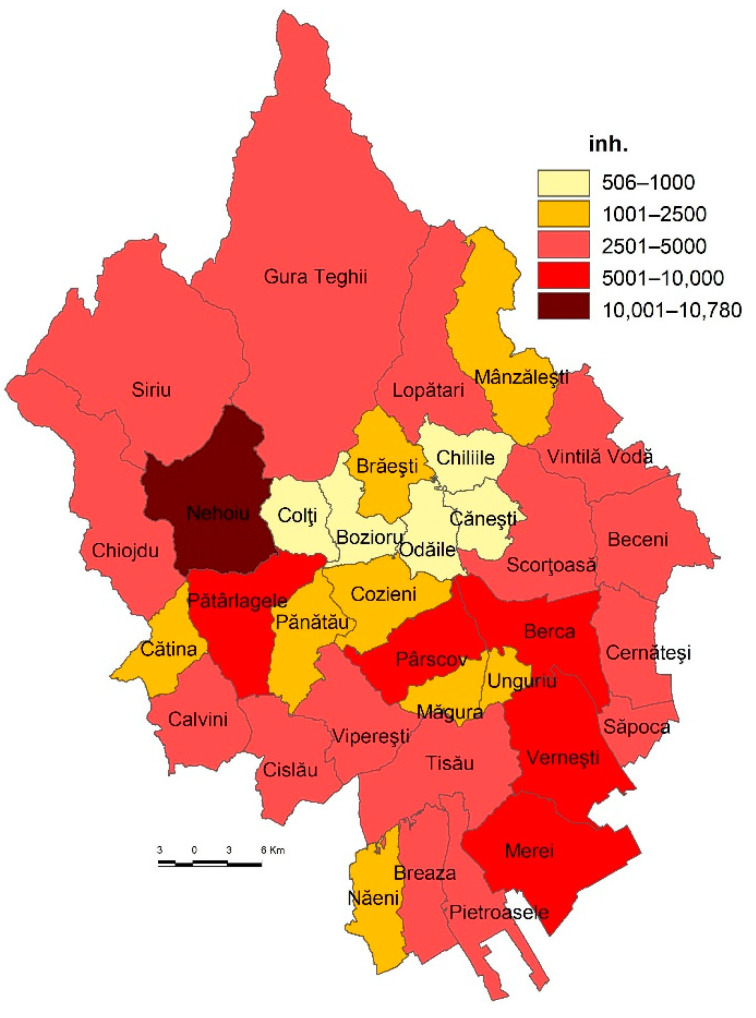
Demographic size at LAU level, 2021.

The study area, a predominantly rural region, is facing complex socio-economic and environmental issues. After multiple socio-economic transformations, as a result of the enforcement of communist policy guidelines and of the post-communist (post-1990) economic, social and political transitions, the study area has faced numerous problems similar to the rest of rural and urban areas in Romania. The latter are reflected in the imbalance between the environmental components, the underdevelopment of the rural economy, and last but not least in the low life quality of the rural communities. In terms of demographic size, the communes are grouped into two main categories: under 5000 inhabitants (28 communes) and between 5000 and 10,000 inhabitants (4 communes). The two towns, Nehoiu and Pătârlagele, are small sized, with a population of 10,492 inh. and 7319 inh., respectively (Figure 2).

The economic activity in the study area is based on the agricultural sector, followed by the tertiary (25.9% of the total employed population) and secondary (20.5%) sectors (National Institute of Statistics, Bucharest, Romania, TEMPO Online). The agricultural sector is mainly made up of vineyards (red wine production), joined by orchards of apples, plums, cherries and apricots. Existing favourable conditions have engendered the possibility of developing the livestock sector, most notably that of the predominance of cattle, pigs, sheep and poultry. Despite the high agricultural potential, the processing capacity of agricultural products is low due to the outdated technologies in use [60]. The secondary sector was affected by the economic restructuring by reducing the over-sized socialist industrial sector, leading to the counter-productive expansion of the agricultural sector. The main industrial activities are related to the wood processing industry (Nehoiu town) and the exploitation of natural hydrocarbon resources (Berca commune). The tertiary sector is dominated by tourism, trade, transport, education and health activities. While the trade and transport activities are rather basic, the health system is characterized by a lack of resources, as well as by an unequal territorial distribution of available resources which makes it difficult for the national and local healthcare system to supply proper treatment and medication in all areas of the country, particularly in rural and small urban areas [61].

The Buzău Carpathians and Subcarpathians are a blend of scenic natural landscapes and rich history valorised through cultural heritage sites, traditional customs and identity items: gastronomy, fairs and markets, religious sites, museums, wine routes, etc. The diversified cultural potential is marked by the presence of the Buzău Land Geopark, Natura 2000, and Siriu and Penteleu sites, integrating an important part of the mountainous area of the study area [62], which set up the unitary conception of development of cultural tourism, among other types of tourism, which capitalized on the two functions of these sites, that is, the protective function on the one hand, and the tourism function, on the other hand.

**Figure 3:**
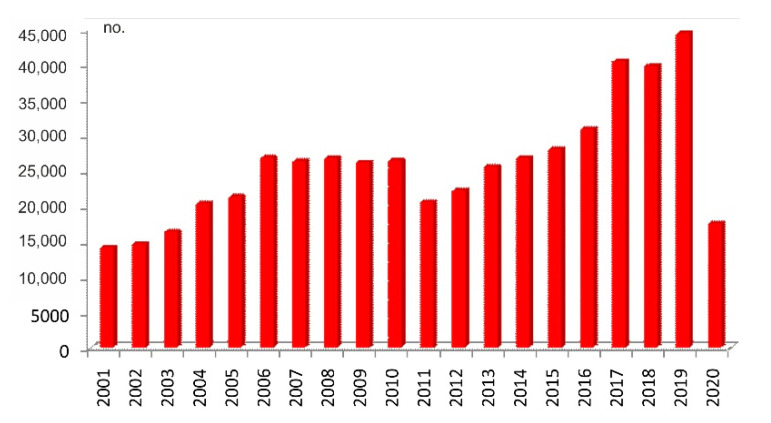
Tourist overnight stays in the reception structures providing accommodation (Data source: National Institute of Statistics, TEMPO Online).

**Figure 4:**
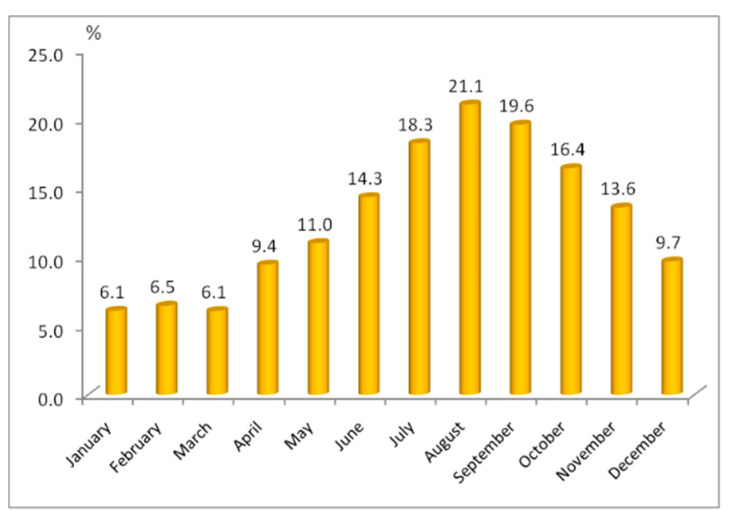
Monthly accommodation occupancy rates (Data source: National Institute of Statistics, TEMPO Online).

The tourist flows dynamic shows a constant increase over the 2011–2019 period, followed by a 59.6% decrease over the 2019–2020 period (Figure 3). According to the National Institute of Statistics, in 2019, Buzău County, of which the study area is a part of, is visited mainly by Romanian tourists (98.98%), the rest being foreign tourists, 79.3% of which being tourists from EU countries. Summer is the busiest season for tourism in the study area (Figure 4) when 47 entrepreneurs mentioned that their businesses are the busiest (94%). Additionally, the school holidays and the autumn months seem to also be busy, while the cold season is not regarded as a busy period for local tourism, as is the case for the spring season.

In terms of the importance of tourists’ origins, domestic tourists rank first—from under 150 km and from over 150 km away—which means that local tourism businesses attract mostly the domestic tourists, the majority of local tourism businesses stating that domestic tourism brings in the highest shares of income.

**Figure 5:**
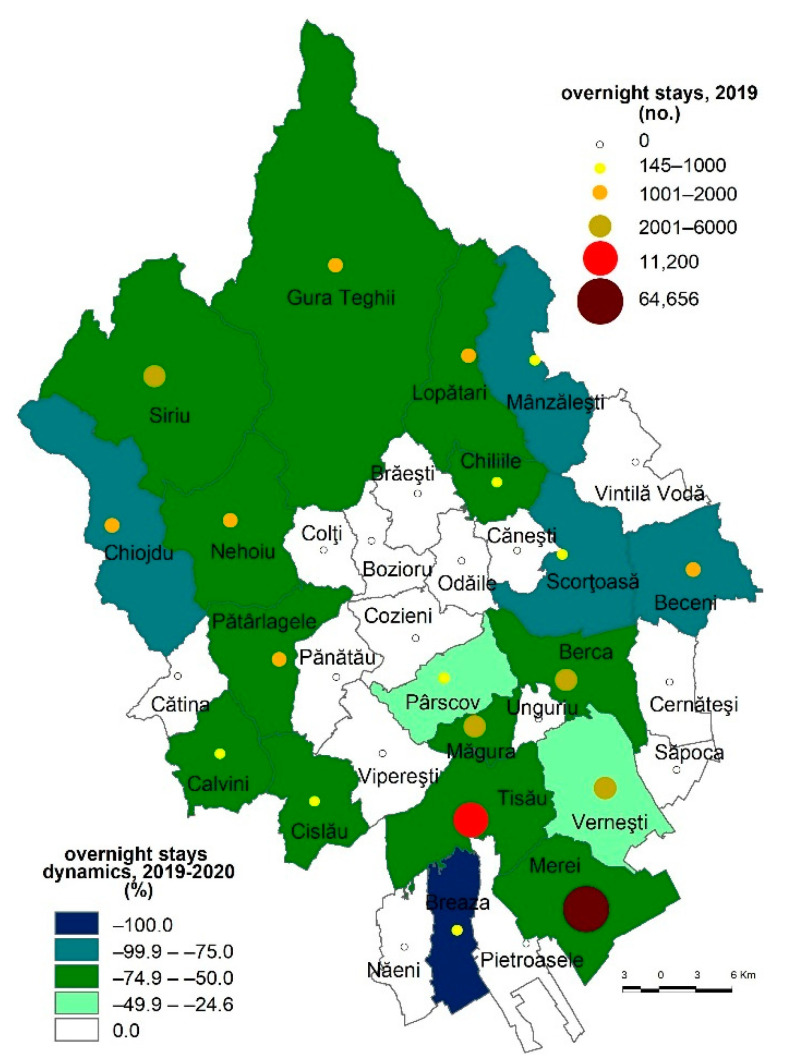
The dynamics of overnight stays, 2019–2020.

Prior to the COVID-19 pandemic, in 2019, the number of overnight stays varied between 64,656 in Merei commune and 145 in Breaza commune (Figure 5).

Within the study area, there are 106 businesses which have tourism activity as their economic profile.

The highest number of tourists registered in Merei commune is due to SărataMonteoru resort, a seasonal spa of local interest where the chlorine–sodium water pool fed by local springs is the main tourist attraction of the area. Weekend tourism is the main type of tourism practiced here, many tourists coming from the Buzău area just for the privilege of using the pools. During the first part of the COVID-19 pandemic (the 2019–2020 period), the overnight stays dynamics shows a decrease by 65.8%, the most significant decline being registered in the Breaza commune (100%), while the lowest decline was found in the Pârscov commune (24.6%) (Figure 5).

## 3. Materials and Methods

The paper was written based on the results of a survey applied to tourists, residents and entrepreneurs/businesses during the field work carried out in the study area in 2020 as part of the framework of the HORIZON 2020 SPOT project. Firstly, the terms tourist, resident and tourism business/entrepreneur were defined in the context of the study area and of the HORIZON 2020 SPOT project: a tourist is an adult who visits the case study area (for one or multiple days) and comes from another region in the country, another country in Europe, or from another continent; a resident is a person who lives in the case study area; and a tourism business/entrepreneur is someone who owns a company connected to touristic area, or is a CEO or (senior) manager at said company.

The targeted interviewed categories as part of the framework of the HORIZON 2020 SPOT project were businesses, tourists and residents. All consortium partners followed the same three surveys related to business, tourists and residents designed by the WP leader. The questions related to the COVID-19 pandemic appeared after the project approval (2019) and were addressed as an extension to the initial scope of the project.

**Figure 6:**
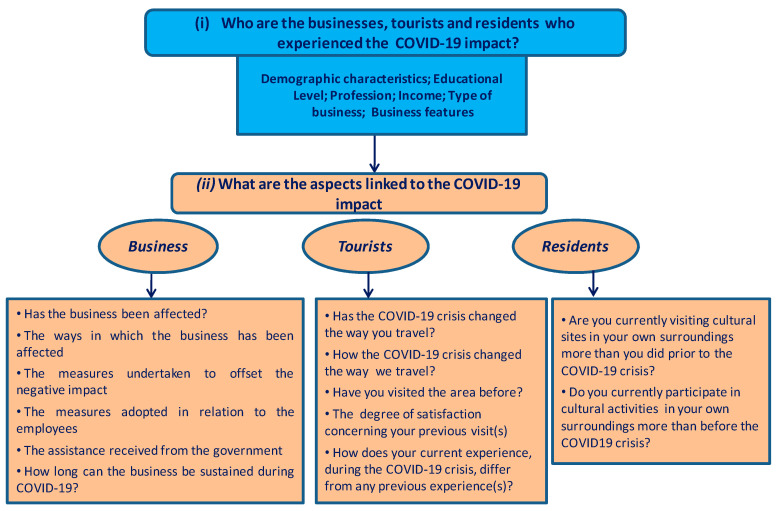
The description of the questions included in the surveys.

The survey offers a qualitative and quantitative picture of the meaning and importance of the effects that the COVID-19 pandemic has had on cultural tourism in the Buzău Carpathians and Subcarpathians. The survey contained two types of questions: (i) who the businesses (e.g., the location of the business, the type of business, the admission fees, the number of visitors, the total visitors’ capacity, the size of the site, the type of visitors targeted, the type of ownership), the tourists and the residents (e.g., gender, age, residence, education, profession, income, household make up) are who were on the receiving end of the COVID-19 pandemic effects and (ii) the aspects linked to the impact of COVID-19 (Figure 6).

### 3.1. The Sampling Method

Sampling is a key issue in survey research [63], whose fundamental step is the identification of the sampling frame. In our case, the sampling frame comprised all the residents, tourists and businesses in the tourism field activity within the study area, as defined in the context of the HORIZON 2020 SPOT project. Sampling itself involved the expert judgement concerning the choosing of the sampling and its size. In the current study, the sampling process was divided according to the general approach, namely the three directions of the study: businesses, tourists and residents, and their challenges stemming from the COVID-19 pandemic.

Thus, in the case of the businesses-oriented field research, the selection of businesses influenced how the entire field research was conducted. Firstly, all businesses in the study area were identified, mapped and grouped into four major categories: (i) accommodation units (e.g., hotels, boarding houses), (ii) restaurants, cafes and bars, (iii) visitor attractions, cultural sites or activities (e.g., cultural heritage sites, vineyards, museums) and (iv) other (e.g., festivals, outdoor activities), in the sense that all accommodation units, restaurants, cultural sites, or cultural events were considered tourist attraction nuclei). They may be associated with the ‘producers’ of cultural tourism [64]. Secondly, the selection of businesses was made based on expert judgment in relation to their locations as it pertains to the main objectives of cultural tourism in the area. It was deemed that each cultural tourism objective should be spatially and functionally connected to at least one category of businesses.

Based on all this, the questionnaires were applied to tourists and residents. The sampling of the businesses was selected based on systematic documentation using internet data sources where the most important accommodation, restaurants, and visit attractions within the study area were presented and ranked (e.g., TripAdvisor, booking.com, https://amfostacolo.ro/, accessed on 2 August 2020); on various blogs promoting local tourism, culture and traditions. In most cases the selected businesses were contacted by phone, then, based on their availability, they were visited on the spot. We tried to approach as many and as varied types of businesses as possible in order to have a complete picture of all the categories of tourist attractions in the area.

The questionnaires were presented to randomly sample tourists via a systematic sampling method (one out of every three tourists, one out of every two business, and one out of thousand households were sampled) to collect the data during daylight hours.

**Figure 7:**
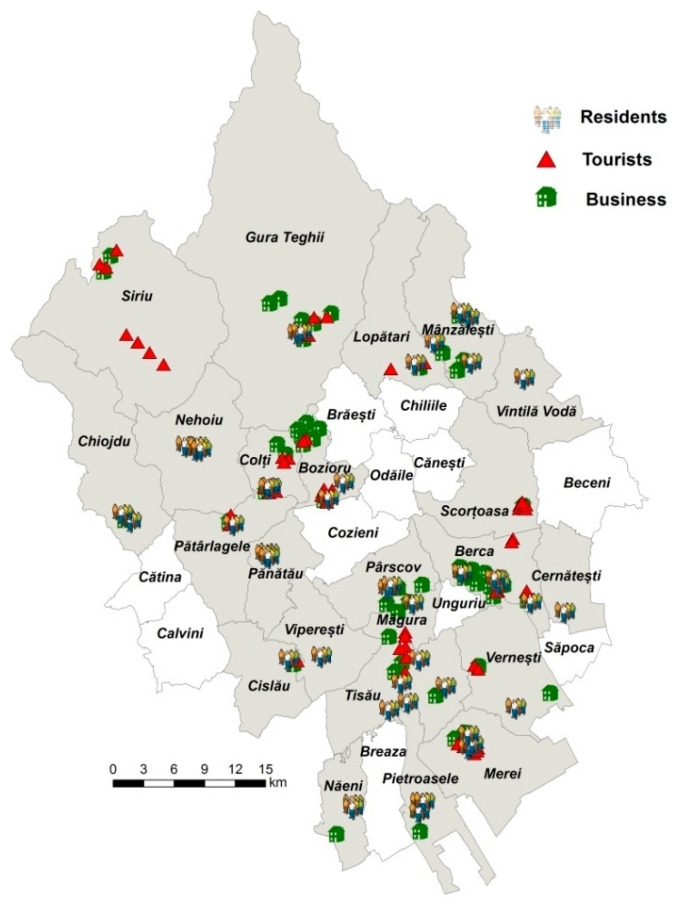
The spatial coverage of the study area by the field campaigns.

The survey had a response rate of almost 94% for business (50 questionnaires out of 53), 91% for tourists (64 questionnaires out of 70), 94% for residents (47 questionnaires out of 50), which is higher than the rates that could be obtained for regular mail or e-mail surveys [65] (Figure 7). These response rates most reflect the use of on-site questionnaires and the data collection assistants who carefully checked the questionnaires to ensure they were properly completed [66].

The tourist-oriented field research. The interviews were taken from tourists found in the proximity of the main tourist attractions and businesses (e.g., hotels, restaurants, museums). The sampling methodology for the tourists’ survey entailed the expert judgement in selecting the interviewed segment. The researchers approached the tourists who were in the proximity of the tourist objectives and of important visitor attractions, sites and accommodation facilities. We tried to reach as many tourists as possible in relation to each business approached or to the main attractions of the region. However, in this randomized process, certain aspects were considered by approaching a variety of tourists in terms of age, gender, professional activity, education, etc. Consequently, it was possible to create an overview of the diversity of tourists’ preferences and perceptions.

As in the case of tourists, when it comes to residents, the questionnaires were addressed to two major categories of residents: (1) residents identified in the localities with tourist attraction sites and tourism-related businesses, where the questionnaires for tourists and for businesses were also applied; moreover, unlike tourists who were mainly sought near tourist attractions or businesses, residents were approached throughout the entire area of the locality; (2) to a lesser extent, residents from remote localities, with reduced accessibility, with few or no tourist attractions or tourist infrastructure; we have chosen to apply questionnaires in these areas in order to understand, by comparison, the perceptions of several categories of residents in relation to cultural tourism in general. We have tried to reach as many residents as possible in relation to their personal relationship with the tourist business or to the main attractions of the region, but also in relation to their personal profile. The result was that it was possible to gain an insight into the residents’ various perceptions and experiences regarding cultural tourism, in general, and the study area, in particular. The overall sampling methodology for the survey was that of randomized selection.

### 3.2. The Sampling Characteristics by Group

The number of minimum interviews was established within the framework of the EU’s Horizon 2020 programme—‘Social and innovative Platform on Cultural Tourism and its potential towards deepening Europeanisation (SPOT)’ taking into account the difficulties arising from the global COVID-19 pandemic.

According to the type of business, 44% of the interviews were taken of the managers of accommodation units, 45% of the representatives of the visitor attraction sites (natural sites, vineyards, museums, cultural heritage sites, theme parks, monasteries, natural sites, farms, horse studs, festivals, children’s workshops, outdoor activities), 6% of the managers of cafés, bars and restaurants, and 5% of other persons (e.g., stores selling local products). The average number of visitors in a year varies from 100 in the case of Plavăţ Donkey Farm to over 100,000 visitors for stores selling local products (Băcănialu’ NeaTicu), the most significant category being that having between 1000–5000 visitors/year with 14 business (56% from the total number) including one theme park, outdoor activities, children’s workshops, religious sites, two museums and cultural heritage sites, three farms/vineyards, and three festivals. Colți Amber Museum, Aluniș Archaeological Site, Buzău Land Geopark and the Mud Volcanoes fall into the category of 10,000–50,000 visitors. The majority of the accommodation units studied, namely 18 cases (81.8% of a total of 22), recorded an average length of stay of guests between 2 and 4 nights. Generally, this is the case of 1-star hotels, guesthouses, B&Bs and hostels (10 cases from a total of 18), but also the case of a few accommodation units classified as being 2-star and 3-star (3 cases) hotels and 4-star hotels (3 cases). Most of the entrepreneurs declared that their business is a family business (18 cases, that is 36% of a total number of 50 businesses), a category closely followed by the private businesses (17 cases, that is 34% of the 50 interviews).

The largest share of tourists interviewed came from other parts of the country (82.8%), while 15.6% were locals (Buzău County) and only 1.6% were international tourists (Poland). The last two categories were significantly lower as a result of the COVID-19 pandemic international travel restrictions. A total of 51.6% of the overall interviewed tourists were men, while the rest (48.4%) were women. Most of the interviewed tourists were professionals (62%) with annual revenues/family predominantly equal or below the national average of EUR 11,000–EUR 20,000 (60%) and under EUR 10,000 (8%). Most tourists fall into the 40–50-year-old age group category (40.63%), as it was the main category of visitors in the Buzău Carpathians and Subcarpathians. It is worth emphasizing that the young visitors category, under 20 years of age, registered a null value, as the study area does not offer diverse possibilities to entertain the young generation. Over half of respondents (51.6%) fall into the revenue category of EUR 11,000–EUR 20,000 and are mainly professional workers, which means the tourists’ income falls into the category of average gross nominal salary in Romania.

Female residents (66%) have proved to be more willing to give their free time to take the interview, followed by male residents (34%). Most of the interviewed residents were adults, between 25–65 years old (83%), followed by seniors, over 65 years old (14.7%) and the young, under 25 years old (2.3%). Most respondents work in professional activities (40.4% out of total number), followed by people which are engaged in services or work in sales (31.4%).

The semi-structured interviews (1) (Table 1), viewed as a quick way of gathering data [67], were conducted face-to-face and contained both open-ended and close-ended questions aimed at revealing the aspects of the CT in its relationship with the COVID-19 pandemic.

The content and structure of the semi-structured interview were driven by the researchers’ intention to make it as exhaustive as possible. Thus, the interview was divided into three parts focusing on: (1.i.) business activity (e.g., types of effects suffered by businesses in the tourist field, the measures applied in response to the COVID-19 pandemic in terms of economic activity and labour force, the perception of the business future in the pandemic context); (1.ii.) tourists (e.g., the changes registered/perceived in the manners, frequency and duration of their trips, the differences between their local cultural tourist experience before and after the COVID-19 pandemic) and (1.iii) residents (e.g., the changes perceived in their participation to local cultural events and as visitors to local tourist attractions).

The authors identified several cases of businesses (3), tourists (8), and residents (5) who have expressed their concern about being interviewed. Thus, they were part of unstructured interviews (2), namely the conversational interview (2.1.), as a flexible interviewing technique [68], which were created on the spot and provided comfort [67,69]. In our case, it is important to mention that, during the conversational interviews, all the issues and questions of the semi-structured interview are followed in detail in the researcher’s mind, he/she considering that a flexible interview ought to follow a structure and a purpose already established in our research [68].

The questions of the semi-structured interview were validated using the expert judgment approach, some of them being selected based on both (1) focus group [70] and (2) nominal technique [71]. (1) For the current study, there were two focus groups involved in the validation procedure: a scientific-oriented focus group convened in June 2021 and a decision-making-oriented focus group convened in June 2021. (1.1) The scientific-oriented focus group was made up of experts in cultural tourism (i.e., Institute of Geography, Romanian Academy, National Agency of the Rural and Ecological Tourism (Buzău Branch). (1.2.) The participants in the decision-making-oriented focus group belonged to several organizations, institutions and local authorities with important roles in the decision-making process in the field of tourism in general, and in those of cultural tourism, in particular: Buzau Land GeoPark, Buzău County Council (through the Regional Development Department and the Buzău County Center for Culture and Art), the Ministry of Development, Public Works and Administration (through the General Directorate for Regional Development and Infrastructure) and representatives of local tourist attraction, accommodation units. For reach consensus, all participants were organized in3 small-groups (A, B and C), based on their expertise in cultural tourism (A), regional and local development through hospitality industry (B) and local administration (C). As a structured variation of a small-group discussion, (2) the nominal technique encourages contributions from everyone prioritising the results. Each participant was asked to comment on the impact of COVID-19 pandemic on each of category: business, tourists and residents, in order to improve the semi-structured interview. The result consists of a set of prioritized impacts that should be reflected by the questions of the three different semi-structured interviews.

## 4. Results

### 4.1. Impact on Businesses

A significant number of businesses (42) were affected by the COVID-19 pandemic, while eight businesses, mainly located in Berca (three cases), Pietroasele (two cases), but also in SărataMonteoru, Nehoiu and Pârscov were not affected. The latter had a mixture of activities which integrated accommodation, visitor attractions and/or sites or activities with farms and/or vineyards and children’s workshops. This multifunctionality of the businesses offered the opportunity to adapt and be resilient when faced with the related health and socio-economic consequences. Another common feature of the businesses that acknowledged that they were not affected by the COVID-19 crisis is their type of ownership: from a total of 8 non-affected businesses, 5 were family businesses, 2 were private businesses and 1 was active in the public sector. By “not-affected by the COVID-19 crisis” we may infer that after the two months’ period of complete shut-down (March and April 2020, during the State of Emergency), business picked up again with just as much dynamism as before, if not more so.

The total average number of Fulltime-Equivalents (FTE) per season working in businesses varies between 489 FTE workers during spring to a maximum of 518 FTE workers during summer. Despite the fact that the highest number of FTE workers is recorded during summer, the differences between seasons are not significant. The main cultural themes offered by these four businesses are sports, biking, trips, hiking, rafting and kayaking, meaning activities which require certain natural and weather conditions, thus being impossible to undertake during cold and/or intermediary seasons (i.e., winter and, respectively, spring and autumn). The FTE’s per season working in businesses during a pandemic year, as a share of the total number, represent high values varying between 83.3% and 86.2%. These high values show that, in terms of labour force occupancy, the impact of the COVID-19 crisis was not entirely consequential, despite the negative effects of the complete shut down during the two spring months of 2020.

**Figure 8:**
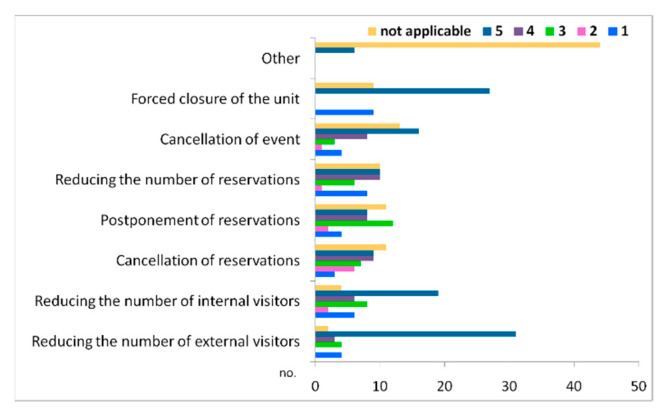
Types of impacts of the COVID-19 restrictions on local businesses.

Our analysis by type of impact of the COVID-19 pandemic on local businesses shows that most entrepreneurs granted the level of disturbance the higher score of “5”, especially referring to the following types of impacts: “reducing the number of external visitors” (31 respondents in the accommodation and visitor attractions category), “the forced shut-down of the unit” (27 cases mainly in the accommodation business) “reducing the number of internal visitors” (19 entrepreneurs) (Figure 8). 

Additionally, 16 entrepreneurs mentioned the “cancellation of events” as a type of negative impact, mainly regarding the visitor attractions and accommodation units. Level “1” is mentioned by fewer entrepreneurs, especially for the types of impacts related to “decreasing the number of reservations” (8 cases) and to “cancelling bookings” (9 cases). 

**Figure 9:**
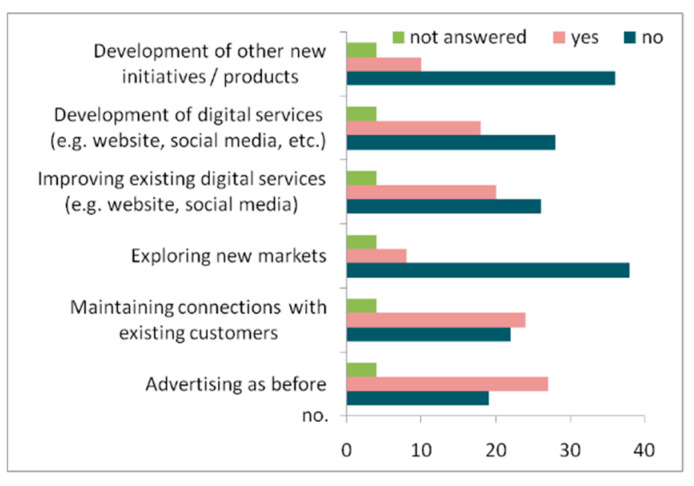
Measures taken to offset the negative impacts of COVID-19 restrictions.

New measures aiming to offset the negative impact of the COVID-19 pandemic have been taken. The respondents have mentioned mainly three measures: 27 respondents (58.7%) mentioned “advertising as before”, 24 respondents specified “maintaining connections with existing customers”, and 20 respondents leaned more on “improving existing digital services (e.g., website, social media)” (Figure 9). The three measures offsetting the negative effects of the pandemic show that the local entrepreneurs are trying to re-use or upload already existing actions, such as advertising, customers’ relationships or the use of existing digital services. Consequently, they are not open to other and/or different/new ways of action in terms of fighting against the negative effects of the COVID-19 pandemic. It is easy to note that the least-mentioned measures were “exploring new markets” (only eight entrepreneurs) and “developing new initiatives/products” (10 respondents). This means that the owners were not creative enough to come up with or develop alternatives to sustain their business reliability.

**Figure 10:**
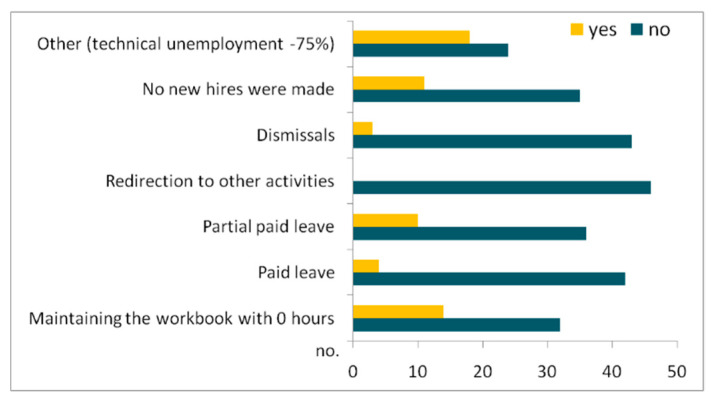
Measures for tourism employees during the COVID-19 restrictions.

New measures were taken regarding employees, among which a relatively frequent one was technical unemployment (18 businesses, mainly from the accommodation category), followed by maintaining the workbook at 0 worked hours (14 cases), partially paid leave (10 cases) and no new hirings (11 cases) (Figure 10). These responses show that local entrepreneurs are trying to maintain the same level of labour force occupancy as before the COVID-19 pandemic, thus preferring several “administrative” measures (i.e., maintaining the workbook at 0 worked hours, technical unemployment) to some drastic measures, such as layoffs (only three cases).

**Figure 11:**
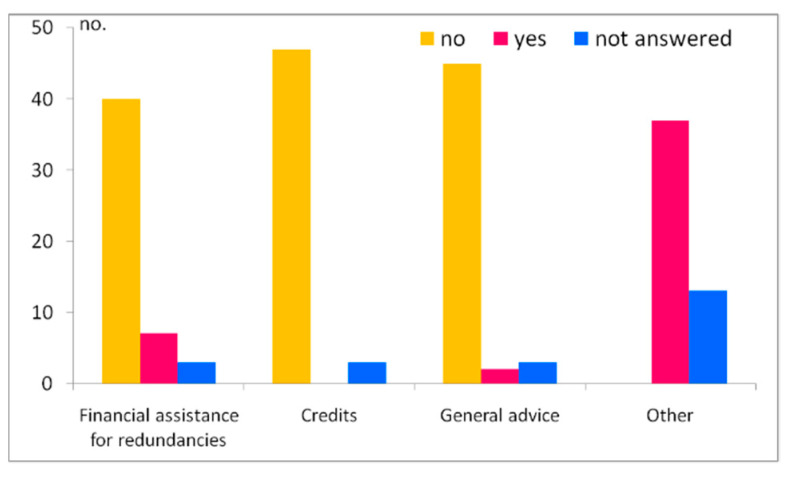
Government assistance to offset the related impacts of COVID-19.

Support under crisis situations is extremely important, but in terms of the assistance received from the government to offset any impact of the pandemic, the great majority of entrepreneurs mentioned that they did not receive either financial assistance for redundancies or credits lines or even general advice (40 cases, 47 and, 45 entrepreneurs, respectively). The category “other” (meaning general advice from the media and technical unemployment at a rate of −75% of a person’s wages) was mentioned by 37 entrepreneurs (Figure 11).

Notwithstanding the support received, or lack thereof, most businesses (95.7%) stated that, given the conditions imposed by the pandemic, they were able to maintain their activity in the local economy for over one year, and much fewer for less, i.e., 3 months (an accommodation business located in Pârscov) and 6 months (an accommodation business located in SărataMonteoru). These results show that local entrepreneurs are confident in their business, at least for the short term (one year). This aspect is an encouraging one, given the context of the COVID-19 pandemic and the employment issues, the measures for maintaining the occupancy level of the labour force, for offsetting its negative effects, and the general lack of government measures for sustaining local economies.

### 4.2. Impact on Tourists

A significant number of tourists have already visited the study area (62.5%). They travelled mainly as a family or group (self-organized or with friends), aged between 30 and 60 years old, falling into the category of professionals with a varying income. They have chosen to return to visit again certain tourist attractions they were impressed with, but also to see new ones or to enjoy other services offered by the area and which have continued to develop more and more in recent years (e.g., gastronomy experiences, wine tastings). The fact that there are also sites of religious pilgrimage is an additional reason to return for a certain category of tourists. The pandemic context is another important factor that made tourists return to the study area as a result of the limitation on the possibility to go on trips abroad or the desire to visit more natural and less accessible areas, away from overcrowding. The fact that a significant number of tourists have chosen to return to the study area soon after having already visited it can be explained by two main reasons: the recent trend to improve the tourist offer by organizing events, gastronomic experiences, wine tastings and the current pandemic context, which, in some cases, makes for a favourable context and responds to the tourists’ desire to find seclusion and peace, close to nature and traditional rural communities. These reasons may also be behind former visitors’ decision to return in 2020 to visit the area again. Following the pandemic, tourists tend to prefer natural, relatively remote, rural and less crowded areas, such as the study area. These particular features have also influenced a fairly large percentage of tourists to visit the area for the first time (37.5%), mainly couples or families between 30 and 50 years of age with a household income varying between 11,000 and 40,000 EUR/year.

**Figure 12:**
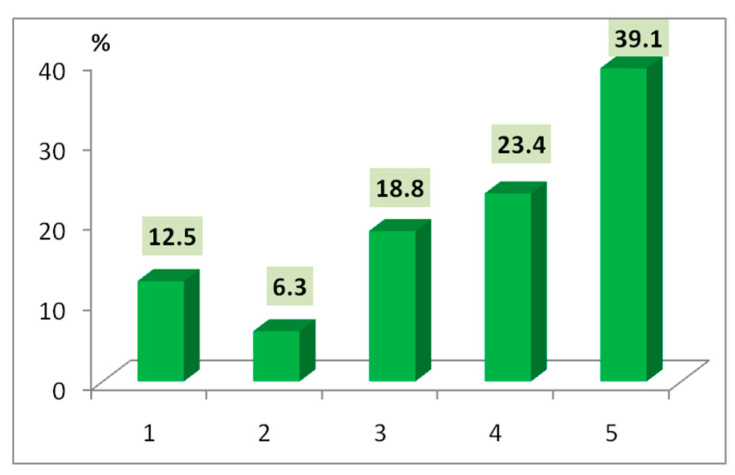
COVID-19 restrictions and travelling behaviour (1—very little, 5—very much).

The COVID-19 pandemic changed the way we travel, as it is coupled with the fact that the largest share of interviewed tourists admitted to being significantly affected as shown by the highest scores attributed: 39.1% (score 5) and 23.4% (score 4). This category included tourists who used to travel often both in the country and especially abroad, young or mature tourists with above average education (tourist, male, 44 years old, high level of education: “I didn’t travel as often as before. And abroad, not at all”). Families with children also used to travel more in the past, but especially in terms of domestic travelling, under certain (child friendly) conditions (tourist, female, 45 years old: “We look for less visited areas and we are very attentive to the safety of our family”). Only 12.5% stated that they were inconsequently affected by the pandemic (Figure 12). They are generally tourists who do not usually travel for their days off (often due to financial reasons) or who practice weekend and/or short-distance vacations, not far from their place of residence.

**Figure 13:**
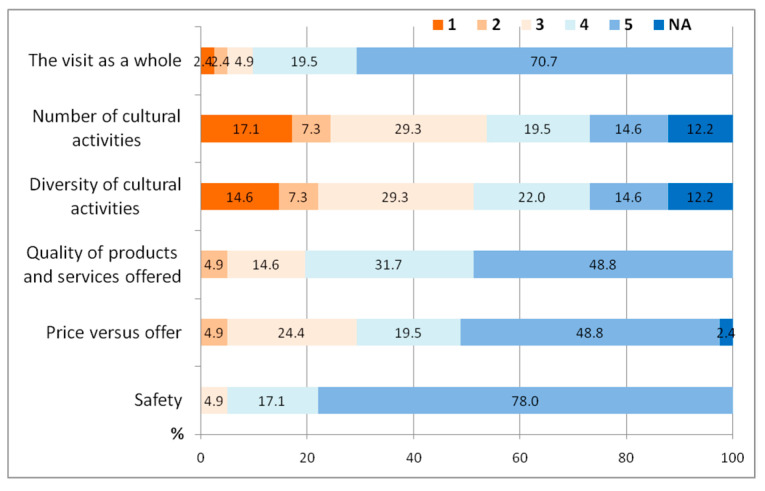
Tourists’ satisfaction during the pre-COVID-19 pandemic visit.

Regarding the satisfaction related to the previous visit, we may notice that safety ranks first for 78.0% (level 5) and 17.1% (level 4), respectively. They are mainly men (59%), older in age, and are the family travelling type. This clearly indicates the impact of the current pandemic on the choice of answers, but also the influence of the high level of safety in tourists’ choosing to return to this area, in this difficult year. A relatively high rating has equally been attributed to the quality of products, services and prices vs. quality, 48.8% (level 5) of the sample included being mainly men, aged 40 to 70 years, professionals, travelling with their families or in groups and having a household income varying between 11,000 and 20,000 EUR/year. These are also decisive factors that determined the return of tourists to this area. Although it received a high level of satisfaction (70%—level 5), the visit as a whole is a relatively abstract indicator, which does not capture all the elements of detail that make up the entire visit (Figure 13). As a result, and a very important one at that, from our point of view, the fact that the diversity and the number of cultural attractions have a relatively low score emphasizes once more the lack of or deficiency in the flagging of tourist attractions, as well as of touristic publicity, advertising and marketing.

**Figure 14:**
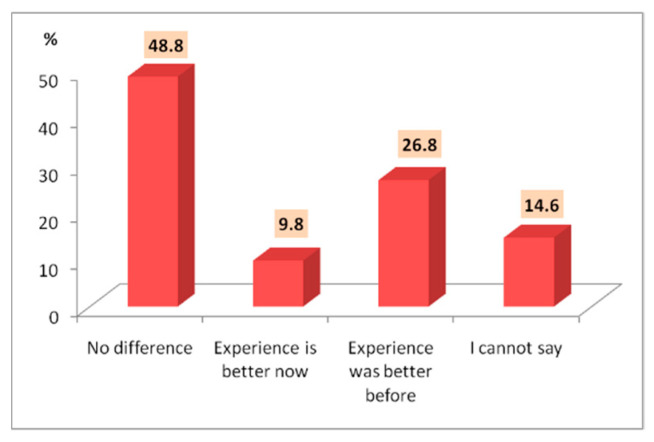
The degree of satisfaction before and during the COVID-19 pandemic.

Some differences in satisfaction before and during the COVID-19 pandemic were registered. Most tourists (48.8%) indicated no difference in terms of their current experience in the area compared to their previous one. This may have a negative meaning which may suggest that not many improvements have been made related to the diversification of touristic offers, infrastructure, the promotion of tourist attractions, etc. This is confirmed by the rather high percentage of interviewed tourists (26.8%) who claim that the experience had previously been better, showing that perhaps either some tourist services or the infrastructure have actually declined over time. This comprises tourists’ families falling into the professional category with a household income of under 20,000 EUR/year. At the same time, this statement most likely takes into account the pandemic context, as tourists felt safer in the past, more able to travel and free from restrictions. Although 14.6% of tourists said they did not perceive any differences between the two experiences, it is important to mention that nearly 10%, mainly women, stated that the experience was better this time around (Figure 14). This statement implies two aspects, either they have identified improvements in the tourist activity in the area, or they feel safer due to the additional sanitary and distancing measures imposed by the pandemic context. Some of them specifically value these additional measures.

**Figure 15:**
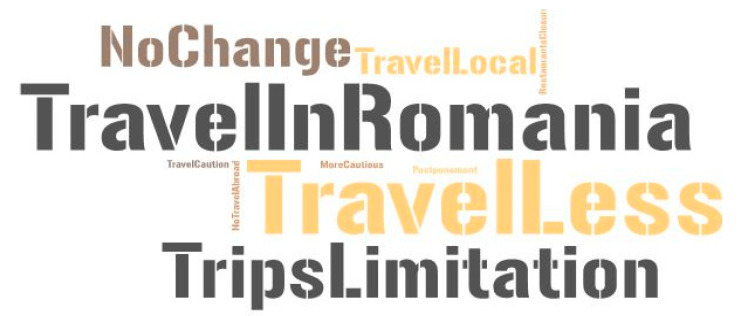
Change in travelling related to the COVID-19 pandemic.

There were different ways in which tourists adapted to new travelling conditions during the COVID-19 pandemic. This question highlighted a number of issues that arose from an increased concern for safety and sanitation, but also related to the restrictions imposed in order to limit the spread of the virus (tourist, female, 42 years old: “I didn’t travel as much as before and, now, I choose accessible destinations according to the regulations regarding the pandemic”). The main and immediate consequences were the limitation of travel in general, but especially regarding travelling abroad (cancellation/postponement of trips), and the shut-down of restaurants and cafés (Figure 15). As a result, tourists opted more for domestic destinations, choosing to spend more time in Romania. Consequently, they began to look for less exploited areas or areas they had not previously visited. This is also the case of the study area, which many of the tourists visited for the first time in 2020, when they began to discover and explore it.

### 4.3. Impact on Residents

**Figure 16:**
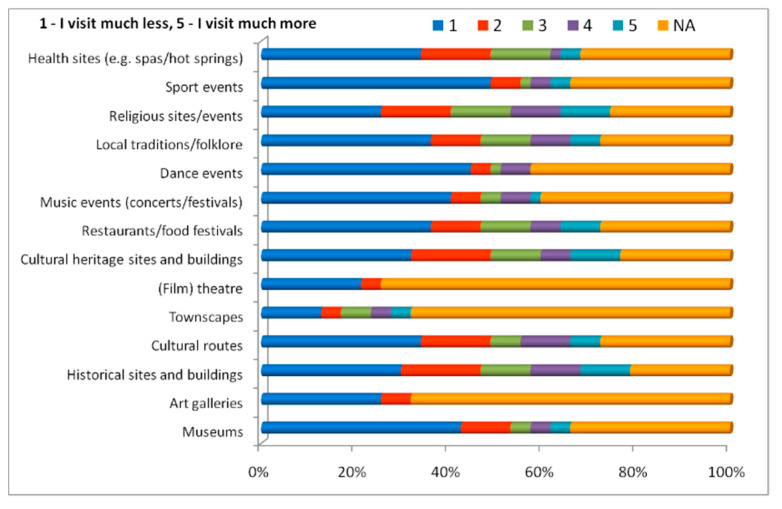
Cultural tourism and COVID-19 restrictions in the eyes of residents.

Within the COVID-19 pandemic context, residents have chosen to visit cultural sites and participate in cultural activities in their own surroundings less than before, as reflected by the field enquiry (Figure 16) and some of the residents did not travel at all to visit different tourist attractions (tourist, male, 74 years old: “I haven’t travelled at all during the pandemic”). Thus, in the case of all cultural attractions, events and sites, residents provided their viewpoint on a scale from “1” (I visit much less) to “5” (I visit much more). An important number of residents ranging from 6 (the minimum value, in the case of “townscapes”) to 23 (the maxim value, in the case of “sportive events”) pointed to level “1” (mainly women over 35 years old, working in services and or as sales workers, with a less than 10,000 EUR income/household/year). 

Level “5” was chosen by five residents from the professional category with two or more members in their household (in three cases, namely “historical sites and buildings”, “cultural heritage sites and buildings” and “religious site/events”). 

Level “3” (I visit the same as before) was chosen by relatively few residents (12.7%). 

A response found very often is “not applicable” which, together with the “1” score (I visit much less) was chosen by the great majority of surveyed residents. There are cases where the number of residents who chose the answer “not applicable” is predominant, reaching 78% for cultural attractions/sites/events, such as “art galleries”, “townscapes” and “film/theatre”. “Music events (concerts/festivals)” and “dance events” were equally considered as “not applicable” and/or assigned level “1” by most residents (almost 42% for each category) is the response to the COVID-19 pandemic restrictions which have also limited their movement. Moreover, visiting “townscapes”, “film/theatre” or “art galleries” are considered an option by residents, given the particularity of the study area or the proximity to areas that might have such tourist offers.

## 5. Discussion

Crises situations (including pandemics) are not new for our planet. Consequently, global tourism has been affected by various events over the years [72]. Many of these crises were generated by events that had a local trigger, thus having effects in local hotspots. However, in the case of the COVID-19 pandemic, both the expression and the impact were felt at the global level. The tourism sector was the most affected segment, with differences from one region to another dictated by the level of tourism development (e.g., over-touristic vs. under-touristic, urban vs. rural) and dependant on the local/regional environmental and socio-economic characteristics. In countries part of the Organization for Economic Cooperation and Development (OECD), in particular, domestic tourism, which accounts for around 75% of the tourism economy, is expected to recover more quickly in the countries, regions and cities where this sector supports many jobs and businesses [22]. Urban destinations, much more dependent on international tourism flows, have been the most negatively impacted by the COVID-19 pandemic [67,73]. Following the pandemic and the imposed restrictions, tourists have changed their behaviour and preferences out of the desire to continue travelling and, at the same time, stay safe. Many tourists are now paying greater attention to low tourist density destinations away from big cities and regions with a lower virus circulation rate [3].

Consequently, the COVID-19 pandemic has shown the need for tourism to change in order to free itself from the immense pressure of mass tourism and make way for new types which are much more adapted to the individual needs of tourists who have reoriented themselves in the post-2020 era towards health, safety, nature and tranquillity. As a result, tourism is undergoing a process of change, both to recover from the economic shocks, and to respond to the new tourist demands. However, models of tourism that destinations want to adopt in the future will also be subject to debate [74]. In many places, this is likely to be a lower volume and higher spending, reversing the decades-old trend of overcrowding and price pressure [13]. Even more than before, governments and tourism industry stakeholders need to consider the costs, risks and impact of global environmental threats on travel and tourism [75]. In addition to the pandemic’s proven negative effects on global tourism, when it comes to local tourism, some nuances ought to be taken into account.

Although there were social and economic losses at the level of businesses and residents, tourists understood the significance of practicing sustainable tourism and its importance for local communities. Thus, they have developed new preferences by putting safety, health and the environment first. This could prompt tourists to opt for safe destinations in the future, such as domestic rural areas [74,76]. This paper shows that diversification was an advantage for local tourism businesses, thus sustaining the outcomes of international studies which conclude that diversification facilitates the increase of resilience [77]. The abundance of literature regarding the pandemic’s impact on tourism has shown the severe effects on businesses, tourists, but also on residents, especially in urban and/or over-touristic areas, e.g., [13,17,18,24,47,57,76,78]. In contrast, in the under-touristic areas, the specific features of the local setting have influenced their ability to become less vulnerable in a different manner. Despite that, tourism scholars have reported a decrease in tourism caused by the COVID-19 pandemic, our rural case-study area shows signs of intensified interests of tourists in travelling after the long periods characterized by travel bans and quarantine regulations. This trend is in accordance with international studies, e.g., [79]. This made the results of the current study provide some unique elements compared to over-touristic regions, where the effects were somewhat similar and predictable. As a result, a general conclusion that emerged from most studies that dive into the COVID-19 pandemic’s impact on tourism was the less frequent use of tourism space while giving preference to places that are outside tourist hotspots [78]. Moreover, people have tended to stay locally and travel within their own countries, which turned out to be inexpensive and affordable compared to international visits [33,76]. The impacts of the COVID-19 pandemic on tourists’ travel intentions indicate the importance of the subjective judgement of danger or economic loss during a potential holiday, the tourists deciding on reducing or even giving up travel plans [47,80,81]. This was no different in the case of Romania, where the study area has become a safe and sustainable alternative to vacations in large domestic resorts or abroad. This paper shows that the cultural and historic heritage owned by the local place have the power to transform the negative pandemic context into a favourable one through the capacity to respond to the tourists’ desire to find uncrowded places, that are close to nature. This idea matches up with studies developed worldwide [74,82]. As a result, and in many ways, the results of the current study are consistent with what has already been shown in literature concerning the impacts of COVID-19 on tourism, while also bringing some particular elements stemming from the combination of the local context and the application of measures and policies at the national level.

According to UNWTO (2022) [2,42], the COVID-19 pandemic brought some changes in customer trends: domestic tourism has shown positive signs on many markets since people tend to travel in their vicinity; travellers go for vacations closer to home; they also believe in the importance of creating a positive impact on local communities; they display an increased search for authenticity; nature, rural tourism and road trips have emerged as popular travel choices due to travel limitations and the quest for open-air experiences. These characteristics were also experienced by the tourism activities in the Buzău Carpathians and Subcarpathians. Various measures to ensure financial support for tourism businesses have been taken by the EU since the beginning of the pandemic. One of the first measures was to provide flexibility under State aid rules to introduce guarantee schemes for vouchers and liquidity support schemes for companies in the Member States through the COVID-19 Response Investment Initiative, but also financing small and medium-sized tourism enterprises (SMEs) through the European Investment Bank [3]. Generally, three types/dimensions of measures ought to be taken for the Buzău Carpathians and Subcarpathians area, as well, as suggested by the United Nations Conference on Trade and Development (UNCTAD) 2021: (1) bringing tourism back on track by restoring the confidence of travellers, who are more concerned about health, and the risk of cancelling their trips; (2) mitigating the socio-economic impacts on livelihoods; developed countries have used fiscal measures to support tourism businesses and workers; (3) making strategic decisions on the future of tourism at the level of each country, deciding what to support and for how long; furthermore, taking into environmental aspects consideration, i.e., increased costs for long-distance flights or the increased social pressure to avoid them.

## 6. Conclusions

Unlike urban regions or over-touristic areas, the study area, which is rather an under-tourist region, has experienced both negative and (potentially) positive effects. Thus, the “stay-at-home” and “social distancing” policies have led to permanent or temporary unemployment, the shut-down of places and businesses, as well as cancellations. Then, the slow relaxation of restrictions opened up new opportunities to capitalize on the new context that reshaped tourists’ behaviours and preferences for more isolated, quiet and safe destinations. In line with the above, the results of the present survey highlighted how the COVID-19 pandemic has affected (i) small businesses during and after the travel restrictions in terms of revenue losses, temporary shut downs, layoffs/technical unemployment; (ii) the tourists’ travelling preferences and sentiments through safer destination choices, risk perception, travel motivation and confidence, the attitude to local/foreign travel and (iii) the residents’ attitudes and behaviour.

From past pandemics, some lessons have been learned, such as practicing responsibility and care for residents and local communities during the chaotic initial stage or preparing the service providers and workers in the tourism industry [58]. However, during the recent COVID-19 pandemic, no tourist destination remained unaffected, be it urban, rural, natural, cultural, mixed, or otherwise with differences depending on the regional or local factors (e.g., environmental, social, economic, cultural, governmental).

Even tourism in predominantly rural areas, such as the Buzău Carpathians and Subcarpathians was affected although it has proven to be more resilient to the crisis (i.e., the COVID-19 pandemic) due to a both naturally and culturally diversified touristic offer. Businesses have suffered the most from the insufficient or lack of government support. In this case, they acted as a barometer for local wellbeing, especially for residents who are more dependent on local tourism activities (as employees of tourism businesses) or by means of the related activities. A very important aspect for the study area was the ability of business representatives to continue their promotion through their own means (i.e., Facebook, Instagram) during the State of Emergency in order to keep in touch with tourists until the tourist flows were restored, to adapt their businesses by providing other services (e.g., accommodation for construction workers, providing temporary isolation facilities for COVID-19 patients, food delivery services, sale services for local products) to compensate for the insufficient or lack of government support. Tourists, on the other hand, have (re)discovered the area or used it as a safe alternative following the restrictions related to long-distance or foreign tourism. After the lifting of the State of Emergency and the relative relaxation of domestic travel conditions, the tourist flow in the study area was higher than in previous years. Tourists were mainly attracted by the naturalness of the region, by the relative isolation from the crowded tourist areas, or by the new sanitary conditions that inspired safety. Thus, many tourists returned to the study area after many years or chose to come in 2020 for the first time as an alternative to travel plans postponed or cancelled due to the pandemic situation.

The COVID-19 pandemic restricted travelling, and tourists have stated that they were “travelling less” and that outbound travel had turned into domestic travel, i.e., “travelling locally” and “travelling in Romania”. These new preferences, dictated by the pandemic context, may be exploited and can even further contribute to the recovery and revitalization of tourism in the Buzău Carpathians and Subcarpathians, as well as to the development of new opportunities that would lead to socio-economic and environmental balance across the area. A category less addressed in the context of tourism-related studies, the residents, was also affected by the pandemic impact, especially those involved in the tourism industry, or whose activity is related to it. The predominantly rural characteristics of the region and the dependence of many economic activities on the pandemic restrictions made the residents significantly reduce their involvement in (cultural) tourism. The temporary or permanent unemployment and the fear of infection made residents travel very little and avoid participating in cultural events, which had already been restricted during the analysed period.

The current study revealed a series of solutions that could help the isolated rural communities in the study area to recover with the help of a more sustainable and resilient tourism sector. All the three main tourism actors analysed (businesses, tourists, residents) ought to understand the new path the tourism sector must tread, which entails a greater adaptability to future shocks, an increased demand for natural destinations, associated with heritage and culture, creative tourism, as well as smart tourism. Thus, despite the region’s limited accessibility to internet services, some businesses survived by maintaining contact with loyal customers (hotels and guesthouses that offered catering services and attractive packages for the new season), or with tourists in general (museums, through virtual tours), or maintaining their interest in the area.

### Limitation and Further Research

Since the study was carried out in the summer and autumn of 2020, the first year of the pandemic when the impact of its effects was still fresh, the authors were able to identify a series of shortcomings which might have influenced the results of the study: (i) the lack of confidence of many of the respondents to participate in the survey as a result of the fear of becoming infected with the virus; business managers had limited access to the facilities, while tourists and residents avoided being approached up close; (ii) the remoteness and limited accessibility (unmodernized roads, lack of internet or GSM service) was time-consuming for the authors who, as a result, had a difficult time covering the area in order to conduct the interviews; (iii) the very location of the tourist attractions and tourism-related businesses scattered throughout the study area and the limited access to good roads has led to difficulties in approaching the participants in order to conduct the interviews; (iv) a higher percentage of female than male residents, which reflects the women’s willingness to respond to the survey and the difficulty of including more foreign tourists in order to have a broader image of the challenges of cultural tourism under COVID-19 pandemic; (v) in the case of residents, the sample selection method does not reflect the general socio-economic structure of the resident population, as a sociological selection method was not used; (vi) it is very likely that the immediate impact of the pandemic influenced the answers of some respondents which were more affected by the COVID-19 pandemic (i.e., businesses) and they might become more emotional about some items in the questionnaire.

Despite these limitations, the current study brings new and useful information about an area where empirical investigation on cultural tourism is scarce, even more so on the challenges the main actors involved in this sector are facing under crisis situations, such as the COVID-19 pandemic. However, these limitations can be overcome through future research. Thus, built on the findings of the current study, follow up research may explore the effectiveness of measures and policies used to support businesses and residents, and attract tourists in the study area during and following the COVID-19 pandemic. Additionally, a future study can use other methods of data collection that may implement extended qualitative and quantitative approaches (mixed methods) aiming to understand the way the study area is ready to tackle any future similar crisis situations.

## Figures and Tables

**Table 1:** Details on data gathering using the interview method.

Type of Interview	Issue/Domains Addressed	Category of Targeted Subjects	Location of Conducted Interview	Period/Interval of Conducted Interview
1. semi-structured	1. (i) business activity	- 50 representatives and/or managers of local businesses from the tourism field; - 64 tourists; - 47 residents;	23 LAU within the study area (Figure 7)	August and September 2020
	1. (ii) tourists			
	1. (iii) residents			
2. unstructured 2.1. conversational interviews	2.1. follows the issues/domains mentioned at 1.(i), 1.(ii) and 1.(iii)	- 3 representatives of local businesses from the tourism field (i.e., cafés, bars); - 8 tourists; - 5 residents who refused to take the semi-structured interview;		

## Data Availability

Not applicable.
